# Development of the Pediatric Quality of Life Inventory™ Eosinophilic Esophagitis Module items: qualitative methods

**DOI:** 10.1186/1471-230X-12-135

**Published:** 2012-09-25

**Authors:** James P Franciosi, Kevin A Hommel, Allison B Greenberg, Charles W DeBrosse, Alexandria J Greenler, J Pablo Abonia, Marc E Rothenberg, James W Varni

**Affiliations:** 1 Division of Gastroenterology, Hepatology and Nutrition, Nemours Children’s Hospital, 13535 Nemours Parkway, Orlando, FL 32827, USA; 2 Center for the Promotion of Treatment Adherence and Self-Management, Division of Behavioral Medicine and Clinical Psychology, Cincinnati Children’s Hospital Medical Center, Cincinnati, OH, USA; 3 Clinical Trials Office, Cincinnati Children’s Hospital Medical Center, Cincinnati, OH, USA; 4 Division of Allergy and Immunology, Cincinnati Children’s Hospital Medical Center, Cincinnati, OH, USA; 5 Department of Pediatrics, College of Medicine, Department of Landscape Architecture and Urban Planning, College of Architecture, Texas A&M University, College Station, TX, USA

**Keywords:** Quality of life, Eosinophilic Esophagitis, Children, PedsQL™, Pediatrics

## Abstract

**Background:**

Currently there is no disease-specific outcome measure to assess the health-related quality of life (HRQOL) of pediatric patients with Eosinophilic Esophagitis (EoE). Therefore, the objective of this qualitative study was to further develop and finalize the items and support the content validity for the new Pediatric Quality of Life Inventory™ (PedsQL™) Eosinophilic Esophagitis Module.

**Methods:**

Multiphase qualitative methodology was utilized in the development of the PedsQL™ EoE Module conceptual model. Focus interview transcripts of pediatric patients with EoE and their parents and expert review were previously used to develop the initial items and domains for the PedsQL™ EoE Module. In the current investigation, utilizing the respondent debriefing methodology, cognitive interviewing was conducted individually with pediatric patients with EoE and their parents on each newly developed item.

**Results:**

Information from a total of 86 participants was obtained in combination from the previous investigation and the current study. From the previous 42 focus interviews, items were developed around the domain themes of symptoms, difficulties with eating food, treatment adherence, worry about symptoms and illness, feelings of being different than family and peers, and problems discussing EoE with others. In the current study’s cognitive interviewing phase, a separate cohort of 44 participants systematically reviewed and provided feedback on each item. Items were added, modified or deleted based on this feedback. Items were finalized after this feedback from patients and parents.

**Conclusions:**

Using well-established qualitative methods, the content validity of the new PedsQL™ Eosinophilic Esophagitis Module items was supported in the current investigation. In the next iterative instrument development phase, the PedsQL™ Eosinophilic Esophagitis Module is now undergoing multisite national field testing.

## Background

Eosinophilic esophagitis (EoE) is a chronic esophageal inflammatory condition with a prevalence that continues to increase [1]. Food allergies are a common cause of EoE, and therefore food restriction is a common treatment [1]. Although patients experience significant disease- and treatment-related sequelae, there is no validated EoE-specific health-related quality of life (HRQOL) instrument to document the impact of EoE on the daily lives of affected pediatric patients. Additionally, current clinical practice in EoE pays little attention to patient HRQOL, instead focusing primarily on histologic and symptomatic improvement outcomes [1].

In order to partly address this significant gap in the empirical literature, we recently utilized the Pediatric Quality of Life Inventory™ (PedsQL™) 4.0 Generic Core Scales to investigate the generic HRQOL of patients with eosinophilic gastrointestinal (GI) disease (EGID, including EoE) in comparison with several other pediatric chronic conditions and healthy controls [2]. Not only did patients with EGID and their parents report significantly lower generic HRQOL than healthy controls, but they reported generic HRQOL lower than pediatric patients with inflammatory bowel disease, epilepsy, type 1 diabetes, sickle cell disease, post-renal transplantation, cystic fibrosis, and obesity [2].

Although the PedsQL™ 4.0 Generic Core Scales assess generic issues common across healthy and ill pediatric populations [3], a disease-specific HRQOL instrument is essential to understanding the particular health issues most germane to pediatric EoE patients from their perspective. In addition, an EoE disease-specific HRQOL instrument would be expected to be more sensitive to detecting change in health status over time within a population of EoE children than a generic scale. To better understand differences in health status within the population of pediatric EoE patients and to enhance the ability to measure the impact of disease-modifying therapies, we previously conducted individual focus interviews of pediatric patients with EoE and parents and solicited medical expert review in the development of the conceptual model and the initial items for a new multidimensional disease-specific HRQOL instrument targeted to pediatric EoE [4]. In the current study, we further utilize qualitative methods, specifically cognitive interviewing techniques, to support content validity and to finalize the items for a quantitative analysis subsequent to field testing.

The FDA patient-reported outcome (PRO) guidance for industry defines content validity as evidence demonstrating that an instrument measures “the concept of interest, including evidence that the items and domains of an instrument are appropriate and comprehensive relative to its intended measurement concept, population, and use” [5]. Furthermore, the FDA has emphasized the critical value of patient input in supporting content validity, noting that “documentation of patient input in item generation as well as evaluation of patient understanding through cognitive interviewing can contribute to evidence of content validity” [5]. Qualitative methods, in particular focus groups/individual interviews and cognitive interviewing techniques, have emerged as the standard methodology for supporting the content validity for new or existing PRO instruments [6-10] and have served as the foundation for previous PedsQL™ Disease-Specific Modules [11-18]. These qualitative methods are consistent with recent FDA guidelines on PRO measures, and as such help establish the content validity of newly developed disease-specific HRQOL items [5]. These iterative steps include: a comprehensive review of the literature, expert opinion, and the patient perspective derived from focus groups/individual interviews (used to develop the conceptual model for relevant disease-specific domains), item generation guided by the conceptual model, and the iterative process of revising the items and item content based on patient cognitive interviewing techniques [6-10]. Previously, Flood and colleagues contributed significantly to EoE PRO development by utilizing expert opinion and cognitive interviews to generate a pediatric EoE symptom metric (the Symptom Questionnaire for Eosinophilic Esophagitis) for use by patients aged 8 – 17 and caregivers of patients aged 2 – 7 [19]. However, this measure was limited by age range and methods of item generation.

Given the lack of an empirically-validated multidimensional pediatric EoE disease-specific HRQOL instrument in the extant literature for patient self-report for ages 5–18 years and parent proxy-report for ages 2–18 years, the objective of the present study was to address this gap in the empirical literature and to describe the qualitative methods, specifically cognitive interviewing techniques, utilized in the further item development and content validation phase for generating the final items for the new PedsQL™ Eosinophilic Esophagitis Module for pediatric patients with EoE.

## Methods

All research for this study was performed in compliance with both the Declaration of Helsinki ( http://www.wma.net/e/policy/b3.htm) and Cincinnati Children’s Hospital Medical Center (CCHMC) ethical guidelines for clinical research, in addition to being approved by the CCHMC Institutional Review Board. Parental permission (informed consent) was obtained from all caregivers and assent from children aged 8 – 17. Informed consent was obtained from patients aged 18.

The validated PedsQL™ module development guidelines, the consolidated criteria for reporting qualitative research (COREQ), and the FDA guidelines on PRO development were used to develop the methodology for item development and content validity [5,16,17,20]. Development of the initial items for the PedsQL™ EoE Module began in 2008.

### Research team

The research team in this study was comprised of five healthcare experts in the fields of Allergy and Gastroenterology with a combined experience of over 30 years researching and treating EoE and two psychologists with expertise in PRO development and methodology (including the developer [J.W.V.] of the PedsQL™). While interviewers were specifically trained in qualitative methodology, all members of the research team developed the interview guidelines and reviewed the transcriptions of the audiotaped interviews.

### Study population

Pediatric patients with EoE 5 to 18 years of age and parents of children with EoE 2 to 18 years of age were recruited from the Cincinnati Center for Eosinophilic Disorders clinic in Cincinnati, Ohio. In the initial qualitative phases of the PedsQL™ EoE Module item development, it was vital to assess EoE-specific concerns and not those related to other co-morbidities. Pediatric participants were restricted to those with a confirmed diagnosis of EoE and without other co-morbidities, including: inflammatory bowel disease, celiac disease, psychiatric disorder, and/or therapy with psychiatric/behavioral medication. Purposive sampling was utilized to ensure that the full clinical spectrum of pediatric EoE phenotypes, ages and symptomatology was represented. Participants were given $45 in gift cards for participating.

### Focus interviews

Draft PedsQL™ EoE Module items were developed from 42 transcripts of 18 child and 24 parent individual focus interviews. Details of the individual focus (concept elicitation) interviews have been described previously [4]. Transcriptions of audio-recorded interview sessions were analyzed by the research team, and participant responses were grouped according to age and subject. Patient and parent responses were separated. The research team reviewed these data to develop domains and initial item content by consensus, with all disagreements resolved after further discussion.

### Expert opinion

Items that emerged from the individual focus interviews were integrated into an initial draft of the PedsQL™ EoE Module [4]. Local (authors) and national (listed in acknowledgements) EoE experts in the fields of Allergy, Gastroenterology, and Psychology then reviewed this draft. To ensure that the focus of the PedsQL™ EoE Module reflected patient and parent perspectives, these clinical experts were allowed to suggest new items, but no items were deleted from the list generated by the patient and parent focus interviews. Several clinical experts suggested adding a number of different questions to address dysphagia, because many patients have adapted to their disease and do not necessarily realize they are having trouble swallowing. Therefore, a question regarding having to drink more liquids when swallowing foods was added to the draft item pool to be reviewed by patients and parents during the cognitive interviewing phase of the study.

### Operationalizing items

The recall period and the Likert-type response scale for the PedsQL™ EoE Module match those of the generic and disease-specific PedsQL™ instruments, which have previously undergone extensive testing [3,11-18]. Items generated by individual focus interview participants and national experts were used to create a draft PedsQL™ EoE Module, utilizing a protocol from the existing methodological literature [21]. The reading level, grammar, and syntax of the PedsQL™ EoE Module items were designed to be structurally equivalent to those in existing PedsQL™ instruments [3,11-18].

### Cognitive interviews

**Figure 1:**
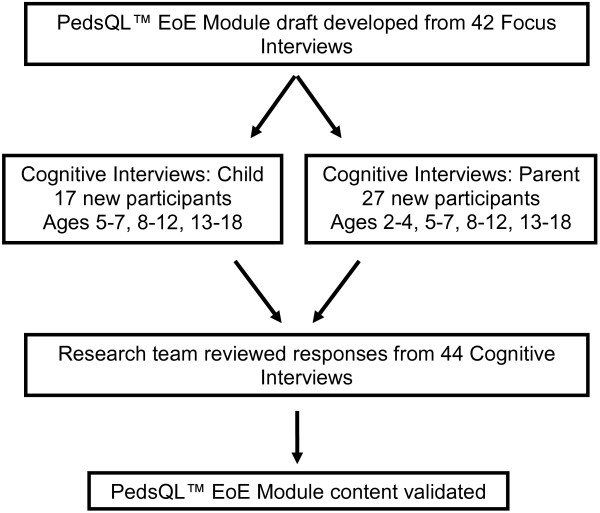
**Focus and Cognitive Interviewing Flowchart.** Focus interview transcripts of pediatric patients with EoE and their parents were used to develop the items and domains for the PedsQL™ EoE Module [4]. Cognitive interviewing was conducted in the current study with separate cohorts of pediatric patients and their parents in the 5–7, 8–12 and 13–18 year old age groups, while parent proxy-reports were also obtained in the 2–4 year old age group.

In the cognitive interviewing phase for the current study, the updated draft PedsQL™ EoE Module was reviewed by a unique cohort of 17 children with EoE and 27 parents of children with EoE who were not participants in the previous focus interviews [4]. Both cohort groups were divided among the patient age ranges of 2 – 4, 5 – 7, 8 – 12, and 13 – 18 years of age, consistent with previous PedsQL™ age groupings. Participants completed the draft PedsQL™ EoE Module instrument and then provided feedback employing the previously described respondent debriefing methodology (see Table 1) [16,17,21]. The goal of the cognitive interviews was to obtain patient perspectives on the meaning and clarity of all aspects of the questionnaire, including: the instructions, domains, questions, and corresponding answer choices. Participants also provided feedback on the overall rationale and disease-relevance of the PedsQL™ EoE Module draft. From these interviews transcribed audiotapes and interviewer notes were used to generate an item-by-item summary of each questionnaire section and included recommendations for item modifications. Utilizing the same methodology as described for the focus interviews [4,21], the PedsQL™ EoE Module items and content were revised. The Flesch Reading Easiness and Flesch-Kincaid Grade Level scores were utilized to further evaluate the reading level and revise item structure as needed. A summary of the content validation methodology using cognitive interviewing is provided in Figure 1.

**Table 1:** Cognitive interview respondent debriefing

**Subject**	**Question**
Directions	How would you make the directions more clear/easy to understand?
	What does "in the past month" mean to you?
	When you see "the past month", what days did you include?
Items	In your own words, what do you think this question is asking?
	What does this question mean to you? What did you think of when answering this question?
	Was this question easy to understand? Are there any specific words that are difficult to understand?
	How would you change the words to make it more clear?
	Was this item hard to answer? If yes, why?
	How did you choose your answer?
Domains	In your own words, what do you think this group of questions is asking about?
	How do you think these items are related?
	Are there any questions that do not belong in this group?
Response Choices	What do you think about the response choices?
	How would you make the response choices clearer or easier to understand?
Overall Assessment	Are there things that we forgot to ask about that you think are important?
	Overall thoughts/opinions of the questionnaire?
	Anything you would change in the questionnaire as a whole?

## Results

### PedsQL™ EoE Module item development: previous focus interviews findings

Basic allergic disease comorbidity and treatment demographics are summarized in Table 2 for this sample of participants in the cognitive interviewing phase. Themes that emerged from the individual focus interview transcripts included symptoms, difficulties with eating, treatment adherence, and discussing EoE with others, as well as worry about the illness and feelings of being different than family and peers [4]. Children with EoE and their parents expressed concern about patients not being able to eat what both their peers and other family members could eat. Pediatric EoE patients often had concerns about trouble swallowing, pain in the chest or abdomen, nausea or vomiting, or difficulties eating. Children described dysphagia in varied ways, including trouble swallowing and avoiding particular foods because of fears these foods would get stuck in the esophagus while eating.

**Table 2:** Demographics and general information for cognitive interview participants

**Basic Demographics, Medical History, & Therapy**	**Among 27 Total EoE patients**
Male	19 (70.4%)
Caucasian	21 (77.8%)
Food Allergies	21 (77.8%)
Asthma	10 (37%)
Eczema	10 (37%)
Allergic Rhinitis	18 (66.7%)
Swallowed Steroid Therapy for EoE	12 (44.4%)
Proton Pump Inhibitor (PPI) Therapy for EoE	15 (55.5%)
Elimination Diet Therapy for EoE	21 (77.8%)
Elemental Diet Therapy for EoE	2 (7.4%)
Child & Teen Interviews	Among 17 Total Child & Teen Interviews
Children 5–7 years old	5 (29.4%)
Children 8–12 years old	6 (35.3%)
Children 13–18 years old	6 (35.3%)
Parent Proxy Interviews	Among 27 Total Parent Proxy Interviews
Parent Proxy Children 2–4 year old children	8 (29.6%)
Parent Proxy Children 5–7 year old children	7 (25.9%)
Parent Proxy Children 8–12 years old children	6 (22.2%)
Parent Proxy Children 13–18 year old teens	6 (22.2%)

Many participants noted that they felt different than their peers, often due to EoE not allowing them to participate fully in social activities centered on food. In addition, the complicated treatment regimens and/or feeding tubes common amongst EoE patients were a burden to them. The subject of communication garnered a wide range of responses as well, including concerns regarding not only health concerns with EoE, but with explaining the disease itself to family, friends, or care providers. The EoE-specific HRQOL domains were distinct from the generic PedsQL™ domains, and included: symptoms, treatment, activities and school, worry, communication, and food and eating. We discovered that patients and parents often had different concerns, illustrating unique but complementary aspects of EoE-specific HRQOL [4].

### PedsQL™ EoE Module item development: cognitive interviewing findings

Basic cohort demographic, age categories, allergic disease comorbidity, medication use and EoE treatments are summarized in Table 2. Selected respondent debriefing results are summarized in Table 3. Most participants thought that the PedsQL™ EoE Module items addressed their EoE-specific HRQOL concerns. Domain-specific patient and parent feedback are presented below.

**Table 3:** PedsQL™ EoE module respondent debriefing results

**General changes**		
The activities and school domain in the first draft PedsQL™ EoE Module were felt to be redundant to the domains in the PedsQL™ 4.0 Generic Core Scales.		
****Symptoms domain****		
Some participants felt that questions were not ordered correctly, forcing them to move back and forth between questions. Question order was changed to reflect these suggestions, resulting in greater clarity. Order of symptoms was changed.		
**Initial item**	**Participant comments**	**Revised item**
I have stomachaches or bellyaches	Although grammatically incorrect, “stomach aches” or “belly aches” was easier for children to understand.	I have stomach aches or belly aches
I don’t grow as much as other kids my age	5 children and many parents suggested removing. A 30-day time frame is not adequate to determine poor growth.	Deleted
****Treatment domain****		
**Initial item**	**Participant comments**	**Revised item**
It is hard for me to take my medicines	5 children felt this question was confusing. Language was changed to determine adherence and emotional functioning in relation to treatment, which tested well.	I do not want to take my medicines
I don’t like going to the doctor or hospital	1 child and 5 parents agreed that degree and type of stress varied between types of visits (i.e. a doctor visit, endoscopy, or allergy testing). The group agreed that separating this question into three separate questions would address this difference.	I do not like going to the doctor
		I do not like getting an endoscopy (scope, EGD)
		I do not like getting allergy testing
****Communication domain****		
**Initial item**	**Participant comments**	**Revised item**
	Several parents wanted to include other adults like teachers, coaches, family members, and babysitters, with whom many children spend much of their time.	I have trouble talking to other adults about how I feel
I have trouble explaining EoE to other people	Many children misunderstood the word “explaining,” so it was changed to “telling.” Parents found their children often do not explain EoE but do tell peers about EoE.	I have trouble telling other people about EoE
****Food & eating domain****		
Concern was raised regarding participants not restricting foods as therapy for EoE, and therefore qualifier question was added, indicating section should be completed only if allergic to foods		
**Initial item**	**Participant comments**	**Revised item**
I eat things I’m not allowed to eat	Several children and parents suggested “sneaking” or “cheating.” This was reviewed with the other cognitive interview respondents who, along with the group, agreed sneaking was a better word. Changed to “allergic” foods to clearly refer to EoE-specific foods, not sugar/sweets.	It is hard for me not to sneak foods that I am allergic to
I don’t want to sit at the table to eat with my family and friends	2 respondents felt that this question was not needed and was not different than other questions in the food and eating domain.	Deleted
****Feelings/worry domain****		
**Initial item**	**Participant comments**	**Revised item**
I worry about eating a food I’m not supposed to eat	4 children thought that referencing allergic foods was easier to understand. Also, parents suggested feelings of being “mad” or “sad” were articulated as concerns that would not be reflected in just “worry.”	I worry about eating foods I’m allergic to or not supposed to eat
		I feel mad (get upset) about not eating foods I am allergic to or not supposed to eat
		I feel sad about not eating foods I am allergic to or not supposed to eat

### Symptoms domain

In the EoE symptom domain, questions concerning dysphagia were asked in several different ways: trouble swallowing, feeling like food gets stuck while eating, needing to drink to help swallow food, and taking a long time to eat. All of these items were felt to be important to participants and were not recognized as redundant. The order of the items was modified per respondent suggestions. A question regarding weight loss and poor growth was felt to be too difficult to answer in the 1 month timeframe and was therefore deleted.

### Treatment domain

Overall this domain was well received by children, teens, and parents. Unlike dietary interventions, respondents expressed clear differences between difficulties with medication adherence and difficulties with taking medications. This item was modified to two separate items that were felt to better capture the intention of these questions: “It is hard for me to remember to take my medicines” and “I do not want to take my medicines.” Adherence and difficulties with nasogastric or gastrostomy feeding therapy were also described as important concerns but were included as conditional questions, because they relate only to children and teens using these therapies. Adherence was not felt to be an important issue for children ages 2 – 4 and 5 – 7, therefore adherence items were not included for these age groups. In the first PedsQL™ EoE Module draft, difficulties with going to the doctor or hospital were described in a single question. Many patients and their parents described clear differences between going to the doctor, undergoing an endoscopic procedure, and receiving allergy testing. The revised module described these concerns as three separate items.

### Activities and school domain

The initial PedsQL™ EoE Module draft included an activities and school domain. Many respondents did not see how this domain differed from a similar domain contained in the PedsQL™ 4.0 Generic Core Scales (School Functioning Scale) and did not feel that these repetitive items contributed to an EoE disease-specific module intended to be co-administered with the generic PedsQL™ instrument. Therefore, this domain was deleted from the final draft of the PedsQL™ EoE Module instrument.

### Communication domain

Communication to parents, friends, other adults, and health care providers about both EoE itself and how children and teens suffering from it are feeling were felt to be important disease-specific concerns. Children, teen, and parent proxy-respondents suggested substituting the word “explaining” with “telling.” In addition, a question was included regarding telling important people with whom children and teens spend a significant amount of time communicating and interacting, such as teachers and coaches, how they feel.

### Food and eating domain

Overall, the items generated regarding food and eating were felt to be significant EoE disease-specific outcomes that reflect important concerns for EoE patients and their parents. However, many patients are not treated with dietary restriction, but instead with only swallowed corticosteroids. Children, teens, and parents who were not restricting foods identified this concern, and therefore this domain was modified to be conditional based on whether dietary restriction was used as a therapy for EoE. Patients without dietary restrictions and their parents are now asked to leave this domain blank. Respondents also expressed difficulty distinguishing the difference between problems following the diet versus problems adhering to the diet. Problems following the diet was captured in the module draft with the two items “It is hard not being allowed to eat some foods” and “I wish I could eat certain things, but can’t,” and the latter question was removed because many children found the two items similar. Questions regarding difficulty not eating the same foods as friends and family were agreed to be separate concerns and clear from the initial instrument draft.

### Worry/feelings domain

Emotional concerns with EoE disease and therapy were nearly universally felt to be important components of disease-specific items. In addition to feelings of worry concerning eating foods, emotions such as “mad” or “sad” were also expressed by families as important aspects to be included. Because these were conditional items (affecting only those with dietary restrictions), this modification was addressed in the revised instrument.

### Final data analysis and construction of the PedsQL™ EoE Module final items

The research team revised items of concern to two or more participants and reached a consensus on all items. In addition, the Flesch Reading Ease and Flesch-Kincaid Grade Level metrics were utilized to modify item language, thereby increasing reading ease, decreasing grade level, and allowing items to be more easily understood by patients and parents. Items were finalized at this stage once content saturation was achieved (no further changes recommended by either the patients or their parents) based on the cognitive interviewing findings.

## Discussion

The PedsQL™ EoE Module is the first and only content-validated pediatric patient self-report for ages 5–18 years and parent proxy-report for ages 2–18 years multidimensional HRQOL measurement instrument specific to EoE, and consequently addresses an important gap in the current empirical literature for this pediatric chronic disease.

Pediatric patients and their parents felt that the EoE-specific domains of symptoms, treatment, worry, communication, food and eating, and feelings better expressed the salient HRQOL concerns of families than the PedsQL™ 4.0 Generic Core Scales alone. The domains of food and worry were frequently identified by patients and families as key components of EoE-specific HRQOL. The use of separate cohorts for the focus interviews and cognitive interviewing phases allowed further elucidation of pediatric patient self-reported and parent proxy-reported opinions and concerns, resulting in content saturation. The cognitive interviews were particularly helpful in clarifying the directions and modifying the items to allow for reading ease by using patient-centered vocabulary. Utilizing the cognitive debriefing methodology in the current study, the semi-structured question format allowed patients and families the opportunity to explain in their own words the impact of EoE on their family and to achieve content saturation prior to finalizing the items.

Although being an important stride forward in the field of pediatric EoE, this study is limited by a few concerns, in particular when applying these results to non-Caucasians and those with co-morbidities. To address these potential limitations, we will next test the PedsQL™ EoE Module items in a large national multisite field test of patients and parents with a wide variety of demographics and comorbidities. Finally, we will then utilize quantitative methods to evaluate the reliability, validity, and responsiveness of the PedsQL™ EoE Module, allowing assessment of the potential performance of the PedsQL™ EoE Module in clinical trials.

## Conclusions

It is of critical importance to include patient self-reported and parent proxy-reported outcomes when evaluating pediatric EoE treatments. Our work both demonstrates and emphasizes the significant impact of EoE on overall health and well-being, rather than just a focus on specific gastrointestinal symptoms, and is an important consideration for future pediatric clinical trials of new medications for pediatric EoE. The PedsQL™ EoE Module will not only address critical gaps in the literature and clinical practice, but also will provide pediatric patients and their families a voice to express the impact of EoE on their daily lives.

## Competing interests

Dr. Varni holds the copyright and the trademark for the PedsQL™ and receives financial compensation from the Mapi Research Trust, which is a nonprofit research institute that charges distribution fees to for-profit companies that use the Pediatric Quality of Life Inventory™.

## Authors’ contributions

JPF substantially contributed to study conception and design, as well as acquisition, analysis, and interpretation of data, drafting and revision of the manuscript, and acquisition of funding. KAH substantially contributed to study conception and design, analysis and interpretation of data, and revision of the manuscript. ABG substantially contributed to study conception and design, acquisition, analysis, and interpretation of data, as well as drafting and revision of the manuscript. CWD substantially contributed to acquisition, analysis, and interpretation of data, as well as revision of the manuscript. AJG substantially contributed to study conception and design, acquisition, analysis, and interpretation of data, as well as drafting and revision of the manuscript. JPA substantially contributed to study conception and design, revision of the manuscript, and acquisition of funding. MER contributed to analysis and interpretation of data, revision of the manuscript, and acquisition of funding. JWV substantially contributed to study conception and design and revision of the manuscript. All authors read and approved the final manuscript.

## Pre-publication history

The pre-publication history for this paper can be accessed here:

http://www.biomedcentral.com/1471-230X/12/135/prepub
